# Cytokinin signaling mediated by KMD2 promotes Arabidopsis hypocotyl grafting success

**DOI:** 10.17912/micropub.biology.001768

**Published:** 2026-02-03

**Authors:** JoonJae Kim, Lucia Strader, Suresh Damodaran

**Affiliations:** 1 Biology, Duke University, Durham, North Carolina, United States; 2 Biology, Salk Institute for Biological Studies, La Jolla, California, United States

## Abstract

Grafting and adventitious root formation are plant regeneration mechanisms used in horticulture. In grafting, a scion (shoot) of one plant is joined to a stock (root) of another plant through vascular reconnection. Adventitious roots arise from above-ground plant tissue, and graft failure occurs when the scion develops adventitious roots. The molecular mechanisms underlying grafting success and adventitious root formation are unclear. We developed a hypocotyl grafting approach to determine regulators involved in grafting success *vs* adventitious root formation. We suggest that reduced cytokinin signaling by the F-box protein, KISS ME DEADLY2 (KMD2, AT1G15670), promotes adventitious root formation over successful grafting.&nbsp;

**Figure 1. Cytokinin signaling through KMD2 is essential for grafting success:**
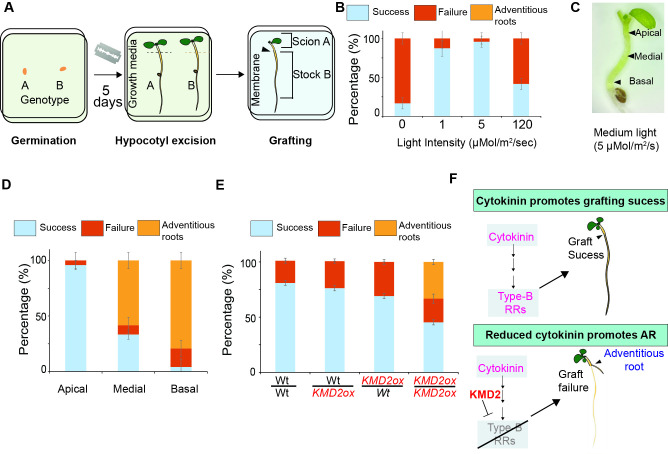
(A) Graphical representation of a modified grafting approach. Seedlings were germinated in PNS for 5-7 days and excised close to the apex (indicated by dashed line) using a double-edged razor blade. Post excision, the stock and scion were aligned (indicated by black arrowhead) for grafting in a new sterile petridish with a wet Hybond N membrane over Whatman filter papers. Grafting success was evaluated after 7-10 days. (B) A stacked bar graph representing the grafting success, failure, and adventitious root formation in wild type (Col-0) seedlings grown under different light conditions and self-grafted. The data represent the average of three replicates, and error bars indicate ±SD. (C) Graphical representation of a semi-etiolated seedling indicating the apical, medial, and basal hypocotyl regions. (D) A stacked bar graph representing the grafting success, failure, and adventitious root formation in wild type (Col-0) seedlings, semi-etiolated, and excised at the apical or medial, or basal region. The data represent the average of three replicates, and error bars indicate ±SD. (E) A stacked bar graph representing the grafting success, failure, and adventitious root formation in wild type/wild type, wild type/ *KMD2 ox* , *KMD2 ox* /wild type, *KMD2 ox* / *KMD2 ox* grafts from semi-etiolated seedlings excised at apical region. The data represents the average of three replicates, and error bars indicate ±SD. (F) Graphical representation of cytokinin role in grafting success vs adventitious root formation mediated by *KMD2 ox* . Cytokinin signaling is essential for grafting success, but reduced cytokinin signaling through overexpression of KMD2 promotes adventitious root formation and reduces grafting success.

## Description

Grafting is a centuries-old horticultural technique used to create chimeric plants by joining the root (stock) of one plant with a shoot (scion) of another plant to improve crop yield and stress resistance (Feng *et al.* , 2024). Grafting success depends on compatibility between the grafting partners. The molecular mechanisms governing whether the grafted plant tissues proceed with graft union formation or shift to an alternative regenerative pathway, such as adventitious root formation, remain poorly understood.

Adventitious roots are shoot-borne roots that arise preprogrammed genetically in some plant species or due to external conditions, such as flooding and wounding. During grafting, the scion can promote adventitious root formation through *de novo* organogenesis instead of vascular reconnection, resulting in unsuccessful graft union formation at the wound site (Turnbull *et al.* , 2002, Melnyk *et al.* , 2015). Both processes share early cellular dedifferentiation mechanisms for either callus formation or direct *de novo* root meristem formation, but require distinct hormonal environments (Bellini, Pacurar et al. 2014;. Auxin and cytokinin are key regulators of tissue regeneration and vascular reconnection during grafting (Melnyk *et al.* , 2015). Yet, the precise molecular switch determining vascular developmental fate at graft interfaces has not been fully characterized. Successful grafting requires balanced auxin levels to promote vascular differentiation (Melnyk *et al.* , 2015), and a higher auxin output is critical for adventitious roots (Sorin *et al.* , 2005, Bellini *et al.* , 2014). Although increased cytokinin sensitivity is known to inhibit the formation of adventitious roots, its role in grafting remains unclear (Melnyk *et al.* , 2015, Damodaran and Strader, 2024).

To determine the regulators involved in grafting success *vs* adventitious root formation, we developed an Arabidopsis hypocotyl grafting approach, using a previously established protocol (Turnbull *et al.* , 2002, Melnyk *et al.* , 2015) (Fig.1A). Grafting is typically performed on seedlings that have been grown under full light conditions (80-120 µMol/m ^2^ /s) (Turnbull *et al.* , 2002). Light conditions alter hypocotyl-borne adventitious root formation in excised hypocotyl (Damodaran and Strader, 2024), and we hypothesized that light levels could impact grafting success. To optimize the light conditions, we grew the seedlings under dark (0 µMol/m ^2^ /s), low (1 µMol/m ^2^ /s), medium (5-10 µMol/m ^2^ /s), and full light (100-120 µMol/m ^2^ /s) (Fig.1B) before grafting. After removing a cotyledon, seedlings were excised close to the shoot apex using a double-edged razor blade and self-grafted to the same seedling (Fig.1A). We found a higher grafting success rate in seedlings grown under medium light / semi-etiolation conditions compared to higher light intensity ( Fig. 1B ).

Distinct gene expression patterns in the hypocotyl region after excision can contribute to regeneration (Damodaran and Strader, 2024). &nbsp;When the hypocotyl is excised at apical, medial, and basal positions, it promotes adventitious root formation close to the cut site, suggesting the regenerative mechanism of the wounded hypocotyl (Damodaran and Strader, 2024). For successful grafting, the hypocotyl is excised at the apical position (Melnyk, Schuster et al. 2015). To determine if the hypocotyl region plays a role in grafting success or adventitious root formation, we excised medium light-grown/semi-etiolated seedlings at basal, medial, and apical regions and self-grafted them (Fig.1C). We observed a higher grafting success when excised at the apical region as previously observed (Melnyk *et al.* , 2015). On the contrary, the grafting success was reduced in hypocotyls excised at the basal and medial regions, which primarily promoted adventitious roots ( Fig. 1D ). This suggested that semi-etiolated seedlings, when excised at the apical region, promote grafting success compared to adventitious root formation.

Cytokinin signaling regulates adventitious root formation near the cut site in excised hypocotyls (Damodaran and Strader, 2024). Reduced cytokinin output due to over-expression of a gene encoding the F-box protein KISS ME DEADLY2, which targets the TYPE B-ARABIDOPSIS RESPONSE REGULATORS, leads to increased adventitious root formation in all regions of excised hypocotyls (Kim *et al.* , 2013, Damodaran and Strader, 2024). We hypothesized that the overexpression of *KMD2* ( *KMD2 ox* ) could alter the grafting success. *KMD2 ox* seedlings grown in medium light and self-grafted at the apical region exhibited low success rates (~45%) and displayed substantial adventitious root formation (~33%) (Fig.1E). Control grafts (Wt/Wt) achieved ~81% success with no adventitious rooting. Heterografts combining *KMD2 ox* and wild type displayed intermediate success rates (~69% and ~76%) with no observed adventitious root formation, suggesting that active cytokinin signaling from stock or scion is sufficient to suppress adventitious root formation and promote graft success in the apical region.

When cytokinin signaling falls below a critical threshold (as in *KMD2 ox* self-grafts), hypocotyl tissues shift toward adventitious root development, potentially altering auxin-to-cytokinin output favoring adventitious root formation (Della Rovere *et al.* , 2013)(Fig.1F). Cytokinin is critical for grafting success but molecular regulators involved in grafting vs adventitious root formation has not been characterized (Feng *et al.* , 2024). The grafting success in &nbsp;Wt/ *KMD2 ox* and *KMD2 ox/* Wt ( Fig. 1E ) further suggests that cytokinin signaling from stock or scion is sufficient for grafting success.

The antagonistic relationship between auxin and cytokinin appears central to this developmental switch, consistent with their established roles in specifying vascular development patterns (Bellini *et al.* , 2014, Melnyk *et al.* , 2015, Feng *et al.* , 2024). Our findings suggest that the molecular switch operates through precise hormone regulation, as demonstrated by the suppression of adventitious rooting in heterografts. The role of KMD2 in cytokinin signaling could be bolstered by measuring temporal and spatial cytokinin levels in the scion and rootstock around the graft union. Thus, we developed a grafting approach that can increase grafting success but also could be used to determine additional molecular regulators that controls vascular reconnection and adventitious root formation (Fig.1F).

Although this approach revealed a potential role of cytokinin regulation of adventitious root formation and grafting success, there are limitations. First, as we used only one KMD2 overexpression transgenic line, and therefore we cannot rule out the possibility of spurious mutations in this line. Our experiments focused exclusively on the Arabidopsis hypocotyl and our findings require further testing in different Arabidopsis tissues and plant species. In addition, the grafting success was evaluated 10 days post-grafting, potentially overlooking early developmental and molecular events that determine grafting success over adventitious root formation.

In addition to developing an efficient grafting approach, the results demonstrated that cytokinin signaling acts as a molecular switch between the two regenerative processes of grafting success and adventitious root formation. This mechanistic framework enables the improvement of grafting success through targeted manipulation of cytokinin signaling in other species.&nbsp;

## Methods

**Plant lines and growth conditions**

Wild type *A. thaliana* ( *Col-0* ) and the overexpression line, *KMD2 ox* *(CaMV35S::KMD2-7)* (Kim *et al.* , 2013), which is in the same background, were used. Seeds were surface-sterilized using 20% bleach for 10 minutes and then washed at least five times with sterile water to remove residual bleach before being resuspended in a 0.1% agar (w/v) solution. The seeds were stratified for 3 days at 4 °C to attain uniform germination and then plated in the plant nutrient (PN) media (Haughn and Somerville, 1986) supplemented with 0.5% sucrose (PNS).

To assay the effects of different light conditions, seedlings were grown under continuous LED illumination at light intensities of 1, 5 and 100 µMol/m ^2^ /s &nbsp;at 22° C in a plant tissue culture chamber (Percival Scientific, model CU-36L4). For the dark-grown seedlings, the plates were covered with two layers of aluminum foil to prevent light.

**Grafting**

Grafting was performed using 5-7 days old seedlings grown on PNS. Seedlings were excised using a double-edged razor blade to make a slant cut at the indicated region of the hypocotyl for both stock and scion. Seven-day-old seedlings were grown on solid PNS medium under controlled conditions (25°C, 16/8 h light/dark cycle) to develop straight hypocotyls suitable for grafting. Following established protocols (Melnyk *et al.* , 2015), transverse cuts were made 0.5 mm below cotyledons using a double-edged razor blade. The grafts were butt aligned on a new petridish with a sterile Hybond N membrane placed above three layers of 3 mm Whatman paper moistened with sterile water. Excision of the cotyledon allowed the grafts to remain in contact with the membrane. Each grafting combination included 12 replicates across three independent trials. Grafted seedlings were maintained under constant light intensity (80 μmol m² s ¹) and then monitored for ten days, categorized as successful grafts (attached grafts with new leaf growth), failed grafts, or scions with adventitious roots.

**&nbsp;**

**Statistical analysis**

The data represents average of three replicates and the error bars represents standard deviation.
